# What do you focus on? An investigation of goal focus from childhood to old age

**DOI:** 10.1007/s00426-023-01804-0

**Published:** 2023-02-21

**Authors:** Lea Moersdorf, Alexandra M. Freund, Moritz M. Daum

**Affiliations:** 1 Department of Psychology, University of Zurich, Binzmuehlestrasse 14, Box 21, 8050 Zurich, Switzerland; 2 University Research Priority Program “Dynamics of Healthy Aging”, University of Zurich, Zurich, Switzerland; 3 NCCR LIVES, Lausanne, Switzerland; 4 Jacobs Center for Productive Youth Development, University of Zurich, Zurich, Switzerland

## Abstract

Goals constitute an important construct in developmental psychology. They represent a central way in which individuals shape their development. Here, we present two studies on age-related differences in one important goal dimension, goal focus, that is, the relative salience of the means and ends of goal pursuit. Extant studies on age-related differences in adults suggest a shift from focusing on the ends to focusing on the means across adulthood. The current studies aimed to expand this research to encompass the *entire* lifespan including childhood. The first cross-sectional study included participants spanning from early childhood into old age (*N* = 312, age range: 3–83 years) and used a multimethodological approach comprising eye tracking, behavioral, and verbal measures of goal focus. The second study investigated the verbal measures of the first study in more detail in an adult sample (*N* = 1550, age range: 17–88 years). Overall, the results do not show a clear pattern, making them difficult to interpret. There was little convergence of the measures, pointing to the difficulties in assessing a construct such as goal focus across a large range of age groups differing in social-cognitive and verbal skills.

**Supplementary Information:**

The online version contains supplementary material available at 10.1007/s00426-023-01804-0.

## Introduction

Goals constitute a widely studied topic in different areas of psychology, including developmental psychology. Goals are often at the center of developmental research, be it in the form of the perception and production of goal-directed behavior in infancy, changes in school motivation in childhood, developmental goals in adolescence, or shifts in goal pursuit across adulthood. This is not surprising, given that goals are hypothesized to drive human behavior by providing direction and meaning, and to represent a central way in which individuals shape their development (Emmons, 1996; Freund & Riediger, 2006; Kruglanski, 1996). However, comparing goals across different phases of the lifespan is difficult because they typically differ on multiple dimensions, such as their temporal scope, content, or motivational orientation. Also, goals are highly subjective and malleable representations and therefore not only differ between persons but potentially also change within a person across time. Moreover, the very definition of goals differs across the literature on child, adolescent, and adult development.

The current studies aimed to investigate whether and how one particular goal dimension, namely goal focus, differs from childhood to old age. To this end, we based our studies on a common goal definition, viewing goals as cognitive representations comprised of means-ends associations (Kruglanski et al., 2002). The dimension of goal focus denotes whether an individual regards the means (process focus) or the ends (outcome focus) of a given goal as more salient at a given point in time (Freund & Hennecke, 2015). Because goals differ individually and are usually embedded in a goal hierarchy, where an end can constitute the means to achieve a higher-level end, it is crucial to define goal focus concerning a given means-end association (i.e., a specific goal). Imagine two people who share the goal of climbing a mountain. One person might concentrate primarily on *how* to achieve the goal (e.g., the climbing technique), the other person might focus on *why* they want to achieve the goal (e.g., to enjoy the view from the top). Whereas the means provide concrete guidelines for actions and are bound to a certain situation or context, the outcomes are represented more abstractly and provide the reason and the general direction for actions (Carver & Scheier, 1998; Freund et al., 2019). Research with adults has shown that goal focus impacts the success of goal pursuit as well as a person’s well-being during goal pursuit (Freund & Hennecke, 2012; Kaftan & Freund, 2018; Krause & Freund, 2016).

Goal focus might differ between persons based on general preferences for certain representations (see Vallacher & Wegner, 1989), between goals within a person (e.g., depending on whether the goal is represented as growth or maintenance goal, Mustafić & Freund, 2012), and change within goals throughout goal pursuit (depending on the phase of goal pursuit, as suggested by Freund & Hennecke, 2015). Additionally, from a developmental perspective, goal focus is hypothesized to change based on cognitive and motivational development (Moersdorf et al., 2022a). Research on adult development suggests changes in goal focus from young to older adulthood, in that older adults increasingly adopt a process focus (Freund et al., 2010). These age-related differences have been documented with different goal-focus measures, such as a thinking exercise or a ten-statements task, which were also used in the current studies. However, as with most psychological constructs, little is known about changes in goal focus and its adaptiveness across the entire lifespan, including early childhood and adolescence (see Wermelinger et al., (2019) for an exception in the field of the perception and production of actions). The primary aim of the present studies was to investigate goal focus from early childhood to late adulthood using a multimethodological cross-sectional approach. Because so far, goal focus has not been assessed across the entire lifespan, we had to develop new goal focus measures suitable for the use with children. To derive our hypotheses, we considered explanations on different levels and from different kinds of literature.

### Hypothesized development of goal focus across the lifespan

As elaborated in more detail elsewhere (Moersdorf et al., 2022a), the developmental literature on action perception, (self-) representations, and motivation suggests that goal focus might undergo multiple shifts across the lifespan. One major hypothesis is that cognitive and motivational development drive changes in goal focus across the lifespan, and that the relative impact of cognitive and motivational processes on goal focus varies across the lifespan. This is not to say that motivational processes do not matter in child development in general, or cognitive processes in adult development, we only refer to the impact of these processes on goal focus. In infancy and toddlerhood, children learn about the effects of actions and build action-effect associations in presence of salient effects (Hofer et al., 2005; Klein et al., 2006). Therefore, a focus on the outcomes might prevail across a multitude of actions (as derived from ideomotor theory, e.g., Greenwald, 1970; James, 1890, and supported by e.g. et al., 2005; Woodward, 1998). During the preschool and kindergarten years (approximately 3–7 years), we assume that the means gradually gain in importance, as children start to become aware of the different ways in which an action can be achieved (as findings on overimitation^1^ and normative criticism^2^ suggest, Keupp et al., 2015; Rakoczy et al., 2008). Around secondary school entry (with approximately 10 years), children’s goal focus might start to shift back towards a stronger outcome focus, because of their increasing ability and tendency to represent abstractly and think about the future, and the school system’s increasing emphasis on tangible outcomes, such as grades (Eccles et al., 1993; Harter, 1999). Two different developmental routes of goal focus are possible during adolescence. On the one hand, adolescents increasingly take long-term consequences into account and need to select future goals, both of which might increase the likelihood of focusing on the outcomes (Nurmi, 2013; Steinberg et al., 2009). Furthermore, adolescents move closer to some important landmark goals (e.g., graduating from school, Nurmi et al., 1994). When important outcomes are approaching, they might become particularly salient, especially when the means to achieve them are obvious. On the other hand, one might argue that a process focus prevails throughout adolescence and only shifts towards an outcome focus with the beginning of adulthood. This idea is based on construal level theory, according to which events in the near future are represented more concretely relative to events in the farther future (Trope & Liberman, 2003). With the nearing completion of adolescents’ landmark goals, they might therefore concentrate on the concrete means to achieve these goals. Thus, based on these landmark goals, opposing hypotheses regarding changes in goal focus are possible during adolescence. Studies on age-related changes in goal focus across adulthood suggest it shifts from a stronger outcome towards a stronger process focus (Freund et al., 2010). This can be explained by changes in the kinds of goals adults pursue: Whereas younger adults are more likely to pursue growth goals (e.g., finding a good entry-level job) relative to maintenance or loss prevention goals, older adults are more likely to adopt maintenance or loss prevention goals (e.g., maintaining one’s health, not losing one’s physical strength, Ebner et al., 2006; Freund, 2006; Mustafić & Freund, 2012). The pursuit of growth goals involves a constant comparison of the actual state with the desired outcome (Carver & Scheier, 1998), likely rendering the outcome more salient than the means. In contrast, when pursuing maintenance goals, the desired state is currently achieved, which should place greater emphasis on the process of how to maintain the current status. This idea has been supported by a study that found an association between goal orientation (pursuit of growth vs. maintenance/loss avoidance goals) and goal focus (Mustafić & Freund, 2012). Additionally, changes in adults’ future time perspective might contribute to changes in people’s goal focus: With increasing age, adults perceive their future time as being more limited (Lang and Carstensen, 2002). This might lead them to concentrate on events that lie in the near future instead of events in the farther future. Together with the idea of construal level theory, this change might lead adults to construe events in more concrete terms the older they grow (Freund et al., 2019; Trope & Liberman, 2003). For adults’ goals, this could imply that they focus on the more concrete, temporally closer means than on abstract outcomes, resulting in an increasing process focus with age.

## Study 1

The first study tested differences in goal focus across a wide age range of 3 to 83 years (split into eight age groups: 3.5–4.5, 6–7, 10–11, 13–17, 18–25, 35–45, 55–65, and 75–85 years). The study investigated the hypothesis that older children and young adults (here the groups from 10 to 25 years) focus more on the outcomes and children before/at the start of school (here the 6–7-year-olds group) and older adults (here the 75–85-year-olds group) focus more on the means (relative to each measure’s neutral point and when testing the groups against each other). For all other age groups, we expected a less clear pattern due to the gradual shifts from outcome to process focus in these age ranges (here the 3–4 and 35–65-year-olds). We did not expect to find sudden shifts at any specific age but instead gradual, broader developmental changes that underlie interindividual variability as well as within-person goal-specific variation.

The study used a multimethodological approach including a variety of behavioral and verbal measures. With few exceptions (i.e., verbal measures), we oriented the assessments of goal focus towards the youngest age group, because their cognitive abilities constrain the feasibility of the tasks regarding the duration and their complexity. This procedure does not come without challenges such as establishing measurement invariance (see Discussion section). Importantly, the previously used measures with adults relied on verbal instructions and written information which are not suitable for young children. Consequently, we had to construct new tasks to assess goal focus across the lifespan. In addition to these newly constructed measures, we included measures that had previously been used for comparison. We predicted that the different measures converge (as indicated by their intercorrelations), with weaker associations between the behavioral and verbal measures, because of non-shared measurement variance.

### Methods

The study was in accordance with the ethical standards of the 1964 Helsinki declaration and its later amendments and approved by the ethics commission of the Faculty of Arts and Social Sciences of the University of Zurich. All participants or their caregivers provided informed consent and were debriefed after participation. No deception was used. Adult participants were reimbursed with 15 CHF (approximately 16 USD) with the option to donate the money to “Doctors without Borders” (https://www.doctorswithoutborders.org/); adolescents received a voucher for the cinema, or a bookstore, and children aged 11 years or younger could choose an age-appropriate toy. The study was preregistered at https://osf.io/jch7m after data from the first three participants had been collected, but not viewed or analyzed. Before the main study was run, a total of nine pilot studies were conducted to remove material biases from the material of the behavioral preference task, to test the items of the goal focus questionnaire, as well as to test the feasibility of the planned procedure. All details on these pilot studies are available on OSF (https://osf.io/qmd8r/).

#### Sample

For the recruitment, we aimed at stratifying the sample with comparable numbers of participants in the following age groups: 3.5–4.5 years, 6–7 years, 10–11 years, 13–17 years, 18–25 years, 35–45 years, 55–65 years, and 75–85 years. Originally, we had planned to include a group of 2.0- to 2.5-year-olds in the study but piloting demonstrated that this age group was not yet able to follow instructions. Furthermore, we had wanted to include adolescents between 14 and 16 years only but had to broaden the age range due to difficulties with recruiting this age group. We planned to collect data from 30 participants per age group.^3^ Because of multiple technical problems during data collection, we had to recruit additional participants. In addition, cooperating with a school resulted in more 18–25-year-old participants than originally planned. Consequently, the final sample consisted of *N* = 312 participants, ranging from 3 to 83 years (see Table 1). Due to different technical problems and the exclusion criteria we applied to some of the tasks, we did not obtain data for all participants for all tasks. Table 1 shows how many participants were included in each task.

**Table 1:** Number of participants, distribution of gender and age per age group (and task)

Age (years)	Total *n*	*n* Female	*n* Male	*M*_*age*_	*SD*_*age*_	*n* Gaze allocation	*n* Imitation choice task	*n* Behavioral preference	*n* Verbal tasks^b^
**3.5–4.5**	**46**	**23**	**23**	**3.50**	**0.51**	**28**	**40**	**43**	**0**
**6–7**	**41**	**19**	**22**	**6.05**	**0.22**	**35**	**40**	**41**	**0**
**10–11**	**32**	**17**	**15**	**10.59**	**0.50**	**29**	**30**	**32**	**32**
**13–17**	**29**^**a**^	**16**	**11**	**14.93**	**1.00**	**27**	**28**	**29**	**29**
**18–25**	**51**	**35**	**16**	**21.00**	**2.34**	**46**	**48**	**50**	**51**
26–34	5	3	2	–	–	5	4	5	5
**35–45**	**30**	**14**	**16**	**40.47**	**2.76**	**28**	**29**	**30**	**30**
46–54	2	2	0	–	–	2	2	2	2
**55–65**	**32**	**16**	**16**	**59.78**	**2.99**	**29**	**30**	**31**	**32**
66–74	7	3	4	–	–	6	5	6	7
**75–85**	**37**	**18**	**19**	**78.24**	**2.58**	**30**	**31**	**37**	**37**
***N* across age**	312	166	144			265	287	306	225

Printed in bold are the age groups we had planned to recruit, 14 participants fell in between those categories, printed in normal font. ^a^Two participants indicated “other” as their gender. ^b^“Verbal tasks” include action descriptions, thinking exercise, and the two motto items

#### Procedure

After providing informed consent, the participants or their caregivers completed an online demographic questionnaire (using the SoSci Survey software; Leiner, 2019). Next, the participants were seated in front of the eye-tracking computer. The first 48 participants were tested with a Tobii T60 eye-tracking computer (Stockholm, Sweden; accuracy: 0.5°, drift: < 0.3°, sampling rate: 60 Hz) at a distance of 60-65 cm between the participant and the screen. Because of technical problems with the Tobii eye tracker, we had to change systems during testing. Therefore, all further participants were tested with an EyeLink 1000Plus eye tracker (SR Research, Canada; accuracy: 0.25–0.5°, sampling rate: 500 Hz) with the display and infrared camera mounted on a movable arm at a distance of 50–55 cm from the participant. We ensured that the visual input the participants received was comparable between eye trackers and programs (e.g., by keeping the visual angle constant that the pictures in the gaze allocation task covered).

Following a nine-point calibration, the participants completed three behavioral tasks in a fixed order. First, they engaged in the gaze allocation task, followed by the behavioral preference task, and the imitation choice task. Initially, we programmed all behavioral tasks in the E-prime 2.0 software (Psychology Software Tools, Pittsburgh, PA). After changing the eye tracker, we gradually programmed all tasks in the Experiment Builder software (provided by SR Research), starting with the gaze allocation task. After the completion of the three behavioral tasks, all participants aged 10 years or older first answered three open-ended control questions regarding the behavioral tasks. These control questions asked whether participants had certain thoughts or looking patterns in the gaze allocation task and what participants based their decisions on in the other two tasks. They were used to detect certain decision biases (e.g., based on object preferences) and served as indicators of face validity. Then participants aged 10 years or older completed online the verbal goal focus measures (programmed with SoSci Survey). The whole session was videotaped for later coding.

### Measures

#### Gaze allocation task

**Fig. 1:**
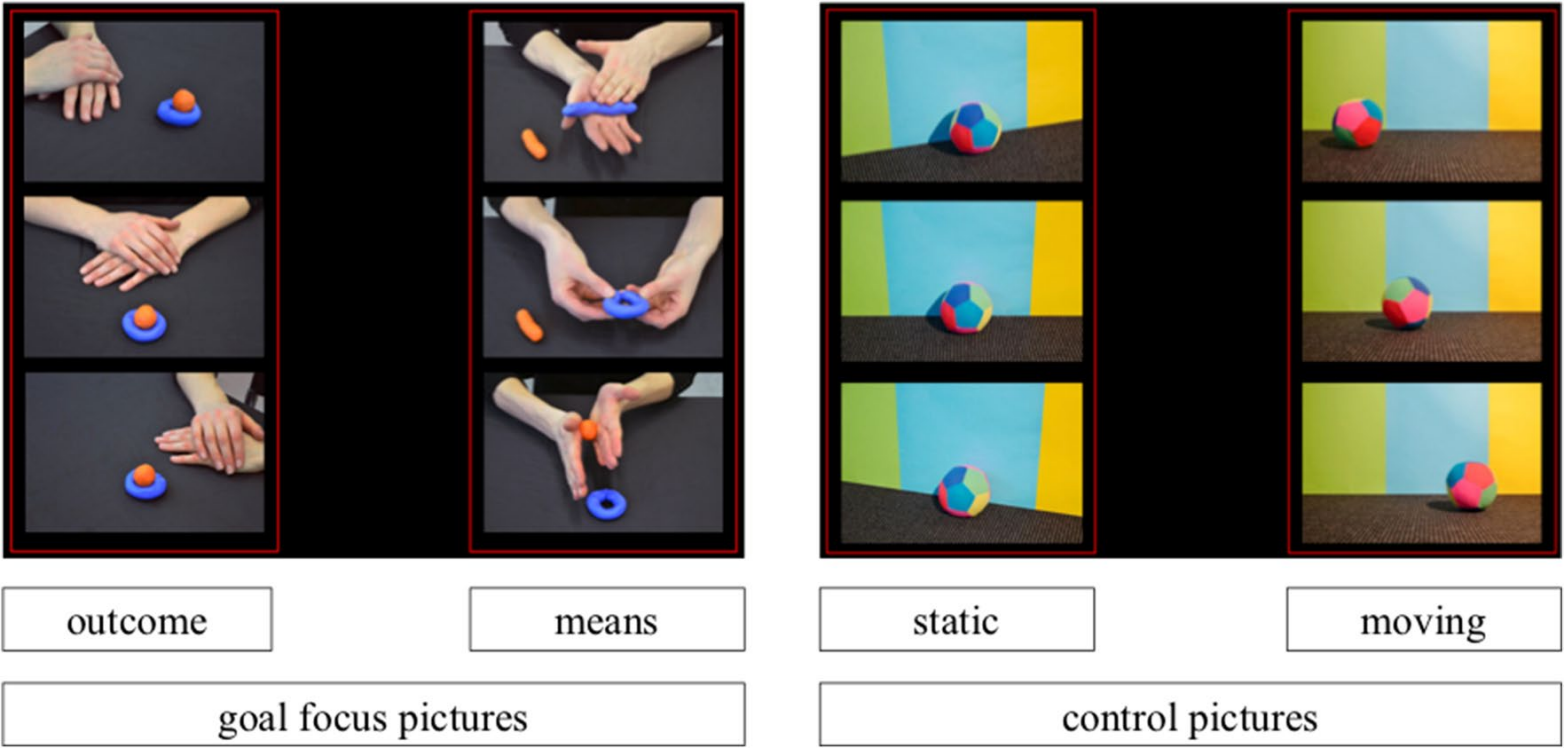
Gaze allocation task: Example of one goal focus trial and one control trial. Note. Goal-focus trials contained means and outcome pictures, control trials contained pictures of static and moving objects

We measured gaze behavior to assess the participants’ focus on the process or the outcome of an observed action based on the allocation of their overt visual attention. The assumption underlying this task was that goal focus as the relative salience of means and outcomes is revealed in behavioral choices and guides visual attention, and was based on the finding that motivational orientations indeed impact overt visual attention (e.g., Nikitin & Freund, 2011). The task consisted of six trials presented on a computer screen and the participants were instructed to “only look at the pictures” without any specific task to perform. In each trial, the participants saw two panels of three vertically arranged pictures, one panel on the left, and one on the right side of the screen. The pictures were of the same size covering the same visual angle (~ 10° × 7°; see Fig. 1). Each trial lasted for 20 s. Of the six trials, three were goal focus trials, and three served as control trials. During goal focus trials, the participants saw one panel of three *means pictures* depicting a human action (e.g., the hand of an actor putting together a playdough figure), and the other panel depicting three *outcome pictures* of that action (e.g., the complete figure) from slightly different perspectives. For the outcome pictures, we used different visual angles to keep the number of pictures constant between means and outcome without showing the identical picture for the outcome three times. This helped to ensure that the outcome pictures contained a similar amount of new information as the means pictures (which varied in content because they showed the progress of an action). The control trials followed the same logic as the goal focus trials but with physical objects instead of human actions: One panel depicted a physical object in motion (e.g., a rolling ball), analog to the human action; the other panel depicted the same physical object (e.g., the ball) static and from three different visual angles (for a list of all objects and motions, see Supplemental Material, Table S1). By comparing the participants’ gaze allocation in goal focus and control trials, we aimed to control for a potential bias towards pictures that imply motion.

Location of the panels (means/motion left vs. right) and order of trials (goal focus vs. control trials first) was counterbalanced, and the order of the three trials within the goal focus and control trials was randomized. For later analysis, we defined two AOIs, one covering the three means pictures, and one covering the three outcome pictures (control pictures: object in motion versus static) similar to the frames depicted in Fig. 1. We defined these two AOIs to be slightly larger than the space taken by the pictures to account for possible inaccuracies during eye tracking (per AOI a total of 25,056 extra pixels, distributed around the pictures as indicated in Fig. 1). The dependent variable was the mean relative time the participants spent looking at the means pictures compared to all pictures on the screen (goal focus pictures score). The same index was computed for the control trials (object in motion vs. all pictures; control pictures score).

### Behavioral preference task

**Fig. 2:**
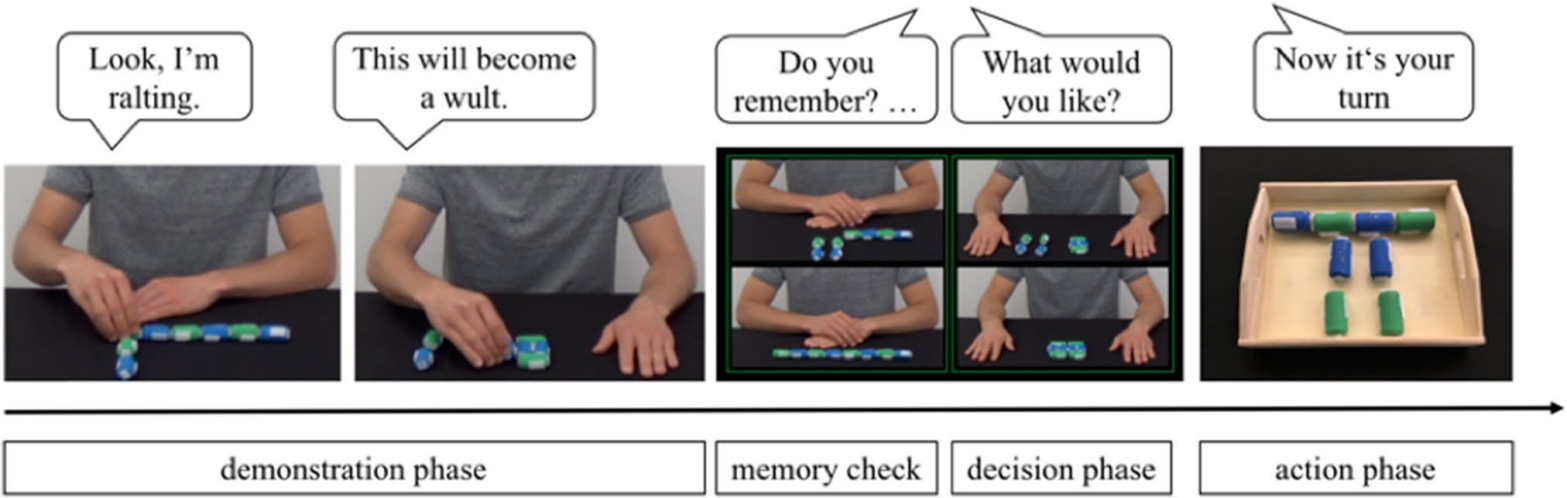
Behavioral preference task: Example of the sequence of events (from left to right). Note. Speech bubbles indicate instructions in the video clip or from the experimenter

A behavioral preference task assessed whether the participants prefer a process or outcome focus when asked explicitly to decide between two alternative actions (means) and objects (outcomes). Similar approaches, such as preferential pointing or choosing, have been used in childhood research to investigate social cognition (e.g., Buon et al., 2014; Hamlin et al., 2011). In these paradigms, one agent usually becomes associated with prosocial behavior and the other agent with antisocial behavior. Afterward, children are presented with both agents, and usually, spontaneously reach or point to one of the agents. This behavior is then interpreted as preference (for a meta-analysis see Margoni & Surian, 2018). In our task, we manipulated the salience of the means versus outcomes in the two options through verbal descriptions (see Elsner & Pfeifer, (2012) for an example of how verbal highlighting can impact the imitation of means vs. outcomes). The participants watched two video clips per trial: One video clip with an action described as a means (i.e., with a pseudo *verb*; “Look, I am *ralting*”; “I have just *ralted*”) and one with the same action described in terms of its outcome, acted on a slightly different object (i.e., with a pseudo *noun*; “Look, this will become a *wult*”; “This is now a *wult*”). All descriptions were based on pseudo words which had been created by changing one letter of Standard German words. Afterward, we presented four pictures depicting the starting and end scenes (unfinished and finished objects) of both video clips. First, the experimenter conducted a memory check with the participants by asking two questions (“Do you remember? Which one will become a *wult*? With which one can you *ralt*?”). Then the participants chose one of the two objects (from one of the two video clips) to act on it themselves (“What would you like?”; “Now it’s your turn”). We added a verbal instruction to the task because we did not expect older children and adults to spontaneously point or reach to one of the options if not instructed to do so. See Fig. 2 for a depiction of the task.

The task consisted of two trials presented in randomized order. Both the object used for the means vs. outcome demonstration and the order of the means vs. outcome demonstration were counterbalanced across the participants. We coded which of the (action-associated) objects the participants chose in each trial (interrater-reliability: *κ* = 1). The dependent variable was the preference score: If the participants chose in both trials the means object, the score was + 1. If they chose the outcome object in both trials, the score was − 1. No preference was coded as 0.

### Imitation choice task

**Fig. 3:**
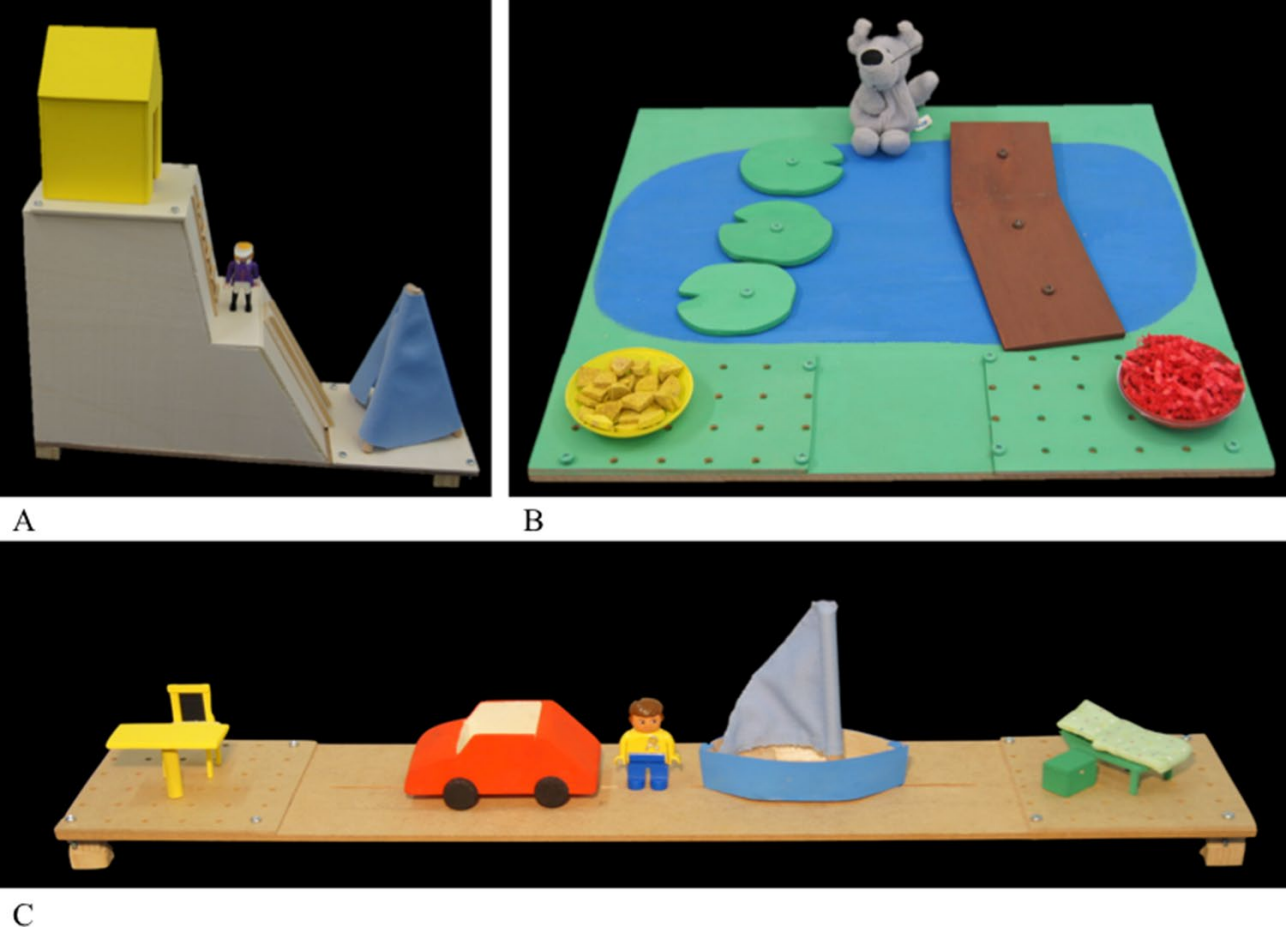
The three apparatus for the imitation choice task. Note. **A** ramp with two paths (up the rungs, down the slide) and two outcomes (house, tent); **B** pond with two paths (lily pads, bridge) and two outcomes (yellow/ red food); **C** board with two means (car, boat) and two outcomes (chair, bed)

An imitation choice task measured goal focus in an imitation setting, in which the participants could choose whether to imitate the means or the outcome. The task (adapted from Elsner & Pfeifer, 2012) consisted of three blocks that were equal in structure but differed in material. The blocks were presented in a randomized order. The different materials are depicted in Fig. 3. Each material comprised two possible paths (means) and outcomes as well as a puppet to act out different directed motion events (e.g., put the manikin into the boat and go to the bed). The location of the means and outcomes could be swapped by the experimenter (with some effort) but not the participants.

**Fig. 4:**
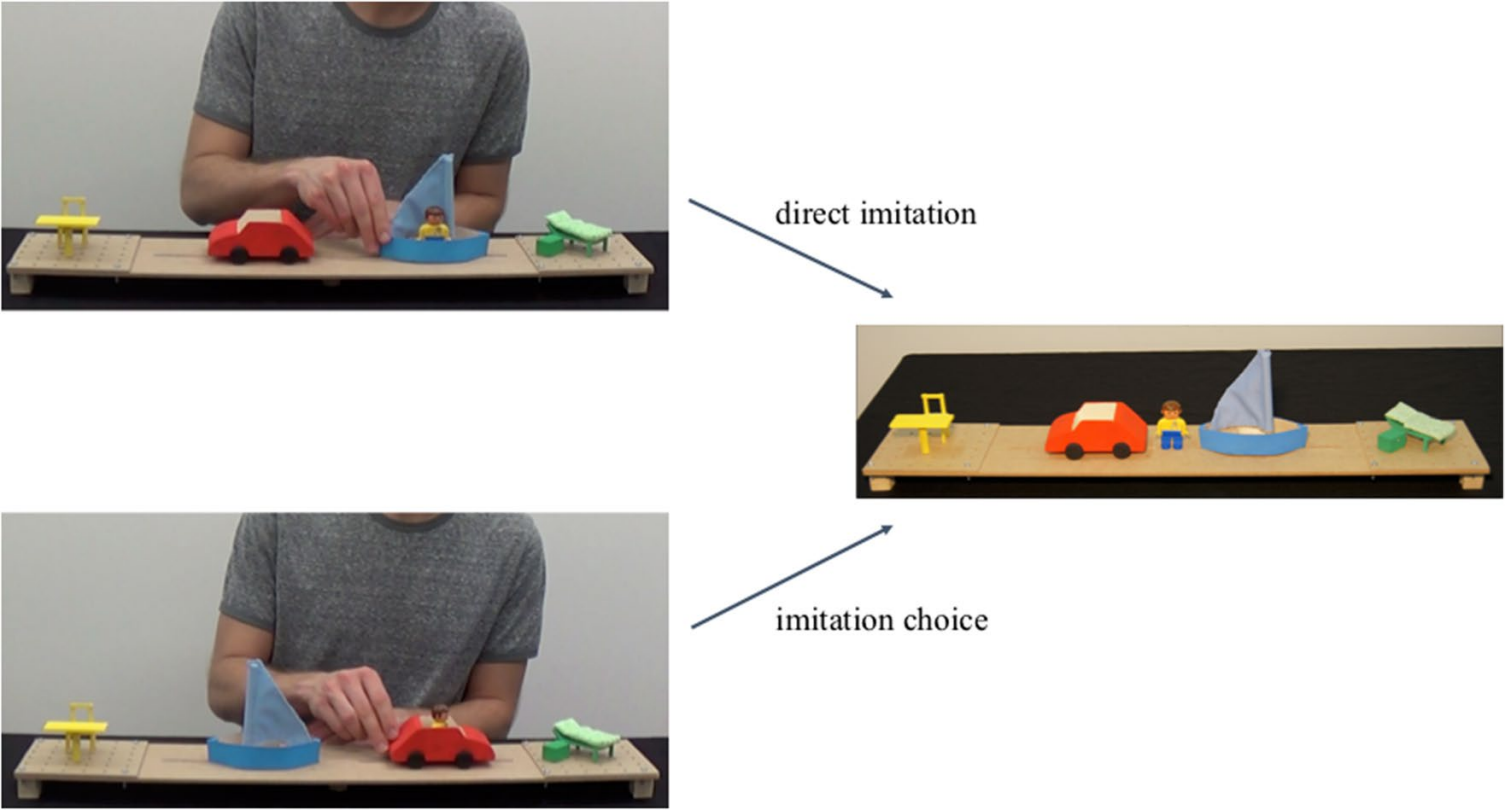
Depiction of a sample direct lmitation and imitation choice trial. Note. On the left side video clip examples are shown for the direct imitation (top) and imitation choice (bottom) phase. On the right side, the material the participants received is depicted. In the upper case, the material looked identical to the material in the video clip. Consequently, the participants could imitate exactly what they had seen. In the lower case, the position of the means (i.e., boat and car) was swapped and, therefore, the participants had to decide whether to imitate the means at the expense of the outcome or vice versa

Each block consisted of a direct imitation phase and an imitation choice phase (see Fig. 4). The direct imitation phase served as a baseline, to test whether the participants spontaneously aimed to imitate the directed motion events from the video clips. During this phase, the participants watched the first video demonstration in which an actor demonstrated a directed motion event with the puppet or manikin (e.g., take the boat and go to the bed; see Fig. 4). Afterward, the participants received the material to act on it themselves (“Now it’s your turn”). Importantly, we did not explicitly tell the participants to imitate what they had seen but only handed them the material. When the participants asked whether they should imitate, we told them that there was no right or wrong way and that they could do as they wished. It was important not to stress imitation because in the imitation choice phase the participants would not be able to imitate exactly what they had seen. This procedure was repeated with the second possible directed motion event with the same material (e.g., take the car and go to the chair; not depicted in Fig. 4). In the imitation choice phase, the participants again saw two directed motion events. This time the material in the video differed from the material the participants received in that either the means or outcomes were swapped (randomized between blocks and across the participants). Consequently, the participants were not able to imitate exactly what they had seen (e.g., take the car and go to the bed; see Fig. 4) but had to decide whether to imitate the means at the expense of the outcome (e.g., also take the car, but go to the chair) or vice versa (e.g., also go to the bed, but take the boat).

We coded the participants’ behavior in the different blocks and phases by determining which components they acted on first with the puppet or manikin (by intentionally touching it with the puppet or putting the puppet in it; interrater reliability: *κ* = 0.99). To be included into further analysis, participants had to imitate the directed motion event (both means and outcome) in at least one of the two direct imitation trials in a block. This served to ensure that the participants were motivated to do what had been shown in the video clips. Furthermore, at least two of three blocks needed to be valid for a participant to be included (i.e., to have results of at least two of three materials). Of all participants with data for this task, six participants did not imitate in any of the blocks, and 13 participants did only in one of three blocks. Consequently, 287 participants were included in the analysis. The dependent variable was the imitation choice score: When the participants imitated the means at expense of the outcome, we coded the trial as + 1. The opposite pattern was coded as -1. Everything else was coded as 0 (e.g., imitating means and outcome or doing something entirely different). Although the participants were not able to imitate means and outcomes *exactly* as was shown in the video clip (because of the swapped positions of means or outcomes), they could use the same means that had been used in the video clip, and then “jump/fly” with the manikin to the other side of the board to the outcome that had been used in the video clip. This behavior was coded as 0. A mean score was calculated across all six trials (three blocks times two trials).

### Verbal assessment of goal focus

The verbal assessment of goal focus was only performed with participants aged 10 years and older. It consisted of three different parts and only included written verbal material, no pictures or video clips. First, the participants read five action descriptions (adapted from Vallacher and Wegner’s action identification questionnaire [Vallacher & Wegner, 1989]), in which simple actions were described with a “how” and a “why” statement (e.g., action = “washing clothes;” how = “putting clothes in the washing machine,” why = “having clean clothes”). The participants chose the statement that, in their opinion, better described the action for each of the five action descriptions. As an indicator of process focus, we counted the number of “how” statements that were chosen by each participant (score ranging from 0 to 5). A similar assessment has been developed by Freund et al. (2010) and used by Krause and Freund (2016), with ten statements for each goal instead of two and more complex goals. These tasks are based on the assumption that “how” statements, which describe the concrete processes by which something is done, are seen as better descriptions by people to whom the processes are more salient in general (as opposed to the “why” statements/the outcomes).

Next, the participants completed the “thinking exercise” as used in Freund et al. (2010): They read the descriptions of the “how thinking exercise,” in which the participants focused their attention on “how one pursues goals” and the “why thinking exercise,” in which the participants concentrated on “why we pursue certain goals.” They chose which exercise they preferred to complete and, based on their choice, either listed two means or two ends of a given goal. The dependent variable was the binary decision. Finally, the participants answered two motto items (“In general, how much are you guided by the motto… a) the path is the goal b) it does not matter how I do it, the main thing is to get to the goal”) on a seven-point Likert scale (from *not at all* to *very much*). We used these two items as separate indicators of process and outcome focus.

### Data preprocessing for the gaze allocation task

We used R (R Core Team, 2018) for most parts of the preprocessing and analysis. Eye-tracking data recorded with the Tobii T60 eye tracker were preprocessed in R, whereas data recorded with the EyeLink 1000Plus underwent the first preprocessing steps in DataViewer (SR Research). Importantly, we held the preprocessing steps as constant as possible between eye trackers. First of all, we made sure that we only considered the time windows of interest for analysis, that is, the six times 20 s of the gaze allocation task during which the participants saw the pictures on the screen. Then, we included a trial only when the participants looked at least 50% of the trial duration (i.e., 10 s) into the AOIs. With this approach, we aimed to ensure that in the included trials the participants had a chance to look at all of the pictures without being distracted (and without necessarily having to look at all of the pictures) and that data was recorded without technical problems. Furthermore, we only included participants in the eye-tracking analysis when they had at least two out of three valid control trials and at least two out of three valid goal-focus trials. We chose this criterion to not rely on data from one specific trial or material, which might bias the findings. Finally, we included only fixations in an AOI that lasted at least 100 ms, because that was the cutoff for the Tobii eye tracker and we wanted to make the EyeLink and Tobii data as comparable as possible.

### Data analysis

We conducted the data analysis in R, mostly with the stats package (R Core Team, 2018). For several reasons we had to deviate from the analyses preregistered on OSF. First, our data did not fit the conditions for a MANOVA (i.e., except for the gaze allocation task, our data were on an ordinal or binary scale). Therefore, we had to resort to non-parametric alternatives (i.e., Kruskal–Wallis tests, chi-square tests, Wilcoxon tests). To test for overall group differences, we ran ANOVAs (for the gaze allocation task), Kruskal–Wallis tests (for the behavioral preference task, the imitation choice task, the action descriptions, and the motto items), and chi-square tests (thinking exercise). When the overall comparisons yielded significant results, we conducted post-hoc pairwise comparisons using Tukey tests and Dunn tests with Holm’s adjustment (FSA package; Ogle et al., 2020). For testing each measure against its neutral value, we ran t-tests (gaze allocation task), Wilcoxon tests (behavioral preference task, imitation choice task, action descriptions, and motto items), and chi-square tests (thinking exercise). Within each measure, we adjusted the significance level according to Bonferroni’s correction to account for the fact that we ran each test for each age group (i.e., behavioral measures *p* = 0.05/8 = 0.006; verbal measures *p* = 0.05/6 = 0.008). To assess the convergence of measures, we used Kendall’s correlations with Holm’s adjustment (psych package; Revelle, 2019) for the associations among all measures except for the thinking exercise. For the thinking exercise, we ran logistic regressions with all other measures as predictors. Additionally, we conducted post-hoc sensitivity analyses for the overall group comparisons, which indicated that we were able to detect medium effects with our sample (effect sizes of *f* = 0.22–0.25 and *w* = 0.25; for details see Supplemental Material, Table S2). Finally, we report inter-item correlations for the items of the gaze allocation task, the imitation choice task, and the action descriptions in the Supplemental Material (Table S3).

### Results

The results section is divided into two parts focusing on two main aspects: First, we present age-related effects, starting with the age-group comparisons and then test each group’s values against the neutral values. Then, we turn to the convergence of measures across all age groups.

We report Bayes Factors whenever possible to provide an estimation of evidence for the null hypotheses (calculated in JASP [JASP Team 2019]; and the BFpack package in R [Mulder et al. 2021]). The Bayes Factors are reported and interpreted according to Schönbrodt & Wagenmakers (2018).

### Age-related effects

#### Gaze allocation task

##### Group comparisons

For the gaze allocation task, we started by taking into account the potential effects of the eye tracker and the differences in implied motion between means and outcome pictures. To this end, we compared a null model to predict the goal focus pictures score to a linear regression model that used the proportion of looking time towards the motion implying pictures from the control trials (control pictures score) and the eye-tracking system (Tobii vs. EyeLink) to predict the goal focus score. The model comparison did not indicate significant differences between the models, *F*(2, 262) = 0.79, *p* = 0.45, and we, therefore, did not consider the control pictures score and eye-tracking system in subsequent analyses.

Regarding age-related differences in the goal focus pictures score, the ANOVA revealed significant overall group differences, although the Bayes Factor indicated no such evidence, *F*(7, 244) = 2.43, *p* = 0.02, BF_10_ = 1.36. Post-hoc pairwise comparisons with the Tukey HSD test did not indicate significant group differences, with the comparisons between the 6–7-year-olds and the 18–25-year-olds (*M* = 0.50, *SD* = 0.07, *M* = 0.55, *SD* = 0.09, *p* = 0.06), and the 18–25-year-olds and the 75–85-year-olds (*M* = 0.55, *SD* = 0.09, *M* = 0.50, *SD* = 0.06, *p* = 0.09) closest to reaching significance.

##### Tests against neutral value

Only the 18–25-year-olds differed significantly from the neutral value after we corrected for multiple testing (Bonferroni adjustment; *t*(45) = 3.85, *p* < 0.001, BF_10_ = 71.74), which indicated that they looked longer towards the process than the outcome images (see Table 2). For a combined scatter plot and boxplot, see Supplemental Material, Figure S1, upper left plot.

**Table 2:** Tests against neutral values by age group

	**3–4**	**6–7**	**10–11**	**13–17**	**18–25**	**35–45**	**55–65**	**75–85**
	Statistic (*p*)							
Gaze allocation	2.65 (0.01) BF_10_ = 3.61	− 0.34 (.74) BF_10_ = 0.19	1.61 (0.12) BF_10_ = 0.63	0.17 (0.87) BF_10_ = 0.21	**3.85 (< 0.001)** **BF**_**10**_** = 71.74**	0.67 (0.51) BF_10_ = 0.25	**2.38 (0.02)** **BF**_**10**_** = 2.17**	− 0.38 (0.71) BF_10_ = 0.21
Behavioral preference	121 (0.84)	176 (0.02)	27 (0.008)	19.5 (0.09)	238 (0.39)	24 (0.39)	69 (0.03)	73.5 (0.19)
Imitation choice	242 (0.68)	336 (0.82)	206 (0.43)	126 (0.08)	586 (0.62)	176 (0.73)	235 (0.01)	334 (0.04)
Action descriptions	–	–	322 (0.26)	301 (0.07)	835(0.10)	294 (0.20)	218 (0.39)	196 (0.02)
Thinking exercise	–	–	0.13 (0.72) BF_10_ = 0.23	0.31 (0.58) BF_10_ = 0.26	3.31 (0.07) BF_10_ = 0.89	**8.53 (0.003)** **BF**_**10**_** = 17.01**	4.5 (0.03) BF_10_ = 2.02	6.08 (0.01) BF_10_ = 4.23
Motto 1	–	–	**416 (< 0.001)**	**302 (< 0.001)**	**815 (< 0.001)**	**292 (0.003)**	**396 (< 0.001)**	**498 (0.001)**
Motto 2	–	–	316 (0.09)	224 (0.09)	**728 (< 0.001)**	184 (0.55)	152 (0.56)	285 (0.14)

Statistic for gaze allocation are *t* values, for thinking exercise *χ*^*2*^, for all other DVs *W* values. Significant results (after correcting for multiple testing) are printed in bold

#### Behavioral preference task

##### Group comparisons

In the total sample analysis, the Kruskal–Wallis test showed significant overall group differences, *χ*^*2*^(7) = 19.30, *p* = 0.01. Post-hoc Dunn tests with Holm’s adjustment indicated significant differences between the 6–7-year-olds and the 10–11-year-olds, *z *= 3.55, *p* = 0.01, and between the 6–7-year-olds and the 55–65-year-olds, *z* = 3.47, *p* = 0.01, in that the 6–7-year-olds chose the process objects more often.

##### Tests against neutral value

None of the age groups differed significantly from zero according to the Wilcoxon signed rank tests after adjusting for multiple testing (*p* < 0.006, see Table 2).

#### Imitation choice task

##### Group comparisons

In the analysis with the total sample, the Kruskal–Wallis test did not reveal any significant group differences, *χ*^2^(7) = 12.53, *p* = 0.08.

##### Tests against neutral value

Similar to the Behavioral Preference Task, none of the age groups differed significantly from zero (Table 2; Supplemental Material, Figure S1). Again, Wilcoxon signed rank tests were adjusted for multiple testing (*p* < 0.006).

#### Action descriptions

##### Group comparisons

The analysis of the total sample indicated significant overall group differences in the Kruskal–Wallis test, *χ*^*2*^(5) = 15.10, *p* = 0.01. Post-hoc Dunn tests with Holm’s adjustment showed significant differences between the 13–17-year-olds and the 75–85-year-olds (*z* = 3.15, *p* = 0.02), and between the 18–25-year-olds and 75–85-year-olds (*z* = 3.03, *p* = 0.03) with the 75–85-year-olds choosing fewer process statements.

##### Tests against neutral value

The Bonferroni adjusted Wilcoxon signed rank tests (*p* < 0.008) did not reveal any values that were significantly different from the neutral value (see Table 2).

#### Thinking exercise

##### Group comparisons

For the thinking exercise, the chi-square test conducted with the total sample did not indicate any significant group differences, χ^2^(5) = 5.71, *p* = 0.34, BF_10_ = 0.01.

##### Tests against neutral value

In the analysis of the total sample, the group of 35–45-year-olds chose the outcome exercise significantly more often than would be expected by chance, *χ*^*2*^(1, *N* = 30) = 8.53, *p* = 0.003, BF_10_ = 17.01, see Table 2.

#### Motto items

##### Group comparisons

The Kruskal–Wallis tests did not suggest any group differences in the motto items; Motto 1: *χ*^2^(5) = 8.15, *p* = 0.15; Motto 2*χ*^2^(5) = 8.90, *p* = 0.11.

##### Tests against neutral values

For the first motto item, all groups differed from the neutral value (*W* = 292–815, *p* = 0.003 ≤ 0.001), indicating agreement with the statement (Table 2, see also Supplemental Material, Figure S1). For the second motto item, only the 18–25-year-olds had a value significantly above the neutral value after we corrected for multiple testing (*W* = 728, *p* < 0.001), indicating an outcome focus.

### Convergence of measures

Kendall’s correlations did not indicate any significant correlations among tasks after we corrected for multiple testing (Holm’s adjustment; see Table 3). The logistic regression with the total sample revealed a significant association between the action descriptions and the thinking exercise (b* = 0.55, *SE* = 0.18, *p* = 0.002, BF_10_ = 9.21), indicating that people who chose more process statements in the action descriptions were also more likely to choose the process thinking exercise (for all predictors, see Table 4). We present the associations among the different measures separately for the age groups in the Supplemental Material, Text S1 and Tables S4-S6.

**Table 3:** Convergence of measures: Results from Kendall’s correlations

Task	Gaze allocation	Behavioral preference	Imitation choice	Action descriptions	Motto 1
Behavioral preference	0.01 (*n* = 262) (*p* > 0.99, BF_10_ = 00.08)	–	–	–	–
Imitation choice	0.03 (*n* = 245) (*p* > 0.99, BF_10_ = 00.11)	− 0.04 (*n* = 287) (*p* > 0.99, BF_10_ = 00.13)	–	–	–
Action descriptions	− 0.01 (*n* = 202) (*p* > 0.99, BF_10_ = 00.09)	− 0.14 (*n* = 222) (*p* = 0.45, BF_10_ = 100.62)	0.02 (*n* = 207) (*p* > 0.99, BF_10_ = 00.10)	–	–
Motto 1	0.01 (*n* = 202) (*p* > 0.99, BF_10_ = 00.09)	0.08 (*n* = 222) (*p* > 0.99, BF_10_ = 00.42)	0.06 (*n* = 207) (*p* > 0.99, BF_10_ = 00.21)	− 0.07 (*n* = 225) (*p* > 0.99, BF_10_ = 00.29)	–
Motto 2	0.07 (*n* = 202) (*p* > 0.99, BF_10_ = 00.27)	0.03 (*n* = 222) (*p* > 0.99, BF_10_ = 00.11)	− 0.08 (*n* = 207) (*p* > 0.99, BF_10_ = 00.39)	− 0.002 (*n* = 225) (*p* > 0.99, BF_10_ = 00.09)	− 0.16 (*n* = 225) (*p* = 0.21, BF_10_ = 490.91)

*p* values are Holm-adjusted

**Table 4:** Convergence of measures: Results from the logistic regression to predict the thinking exercise from the other tasks

Gaze allocation	Behavioral preference	Imitation choice	Action descriptions	Motto 1	Motto 2
− 0.02 (0.16) (*p* = 0.92, BF_10_ = 0.08)	− 0.16 (0.17) (*p* = 0.36, BF_10_ = 0.12)	− 0.21 (0.16) (*p* = 0.19, BF_10_ = 0.18)	**0.55 (0.18) (*p* = .002, BF**_**10**_** = 9.21)**	0.31 (0.18) (*p* = 0.08, BF_10_ = 0.35)	0.14 (0.17) (*p* = 0.41, BF_10_ = 0.11)

Standardized regression coefficients with SEs in parentheses. Significant results (after correcting for multiple testing) are printed in bold

### Discussion

The current study aimed to empirically investigate goal focus across the entire lifespan. We applied multiple behavioral and verbal measures to obtain a full picture of goal focus in a wide age range and across different measures. The current study provided no coherent evidence for systematic differences in process and outcome focus between childhood, adolescence, and across adulthood. Only a few group comparisons yielded overall significant results, and post-hoc pairwise comparisons mostly did not reveal significant differences. If they did, they were partially in the opposite direction than expected. When comparing each age group’s values against the respective measure’s neutral value, only one of the motto items showed significant results in more than one of the age groups. Because some analyses were close to reaching significance, we infer that our results are not clear null findings, instead, they are inconclusive. This was also supported by Bayes Factors around 1, with some exceptions in Kendall’s correlations, where small Bayes Factors suggested no associations.

The lack of support for age-related differences in the thinking exercise was particularly surprising because this is a measure of goal focus where at least tendencies in the expected direction have occurred in previous studies (Freund et al., 2010). One reason why we did not find age-related differences in this study might be that the design influenced the participants’ responses: By the time they completed the thinking exercise, they had already participated in all of the behavioral tasks including the open-ended control questions. This was not the case in the study by Freund et al. (2010) where the thinking exercise constituted the only goal-focus measure. Even though we do not know how this should have impacted the participants’ replies in an age-differential manner, we cannot rule out this possibility. Alternatively, the age-related effect of goal focus might not be as robust as, or weaker than previously assumed. In case of a weaker effect of goal focus than expected, it might be that some of our measures were not sensitive enough to detect these age-related differences. For instance, the imitation choice task or the thinking exercise might very well measure larger differences in goal focus but maybe no subtle changes. In a similar vein, interindividual differences or goal-specific effects might be larger than expected and therefore concealed age-related differences. Furthermore, this study encountered certain challenges during data collection: First, the eye-tracking system had to be changed while data collection was already ongoing. This resulted in partial data loss and rendered the data less comparable. Second, we did not achieve exactly the sample we had hoped for. Whereas it was difficult to recruit adolescents, we ended up with more young adults than planned because of a collaboration. Although we do not know how this should have biased our data systematically, it adds overall noise to the data and thus makes it more difficult to detect effects.

#### Measurement of goal focus

The current study employed a multimethodological approach of measures that did not converge. This casts doubt on the reliability and validity of these measures. Only the association between action descriptions and thinking exercise was significant, indicating that we did not measure a global goal-focus construct across all instruments. Thus, one major concern of the current study pertains to the problem of measurement construction, questioning whether we assessed the same construct across measures. In a similar vein, it is possible that due to our approach to orient our measures toward the youngest age group but to apply them to all groups, the measures were not equally suitable for all groups. Due to the low number of items per measure and small sample size per age group, we cannot offer an analysis of measurement invariance in this study. Related to this is the question of internal consistency of the items within each measure. Due to the low number of items per measure and their response format, we did not report Cronbach’s alpha but instead relied on inter-item correlations (depending on the measure based on Pearson or Spearman correlations, or the Phi coefficients). As suggested by Clark and Watson (1995), inter-item correlations should lie between 0.15 and 0.50, depending on how broad or narrow the measured construct is assumed to be. In this study, the inter-item correlations of the gaze allocation and imitation choice task were very low (see Supplemental Material, Table S3), suggesting that the individual items of these measures do not capture the same overarching construct across the whole sample. This looked different for the action descriptions, which showed an average inter-item correlation of 0.315, indicating a certain overlap between items. Consequently, not all of the applied measures seem to be reliable and therefore cannot be valid.

Additionally, some of the few significant results pointed in the opposite direction than expected based on previous results (Freund et al., 2010). For instance, in the action descriptions, older adults focused more on the outcomes than younger adults (i.e., by choosing fewer process/more outcome descriptions), and in the gaze allocation task younger adults looked more towards the process than outcome pictures (see also the exploratory analyses of linear age-related effects across adulthood in the Supplemental Material, Text S2). Results varied across measures. Whereas we found an association between the thinking exercise and action descriptions, there was little sign of convergence among the behavioral measures or between behavioral and verbal measures, in many cases, the Bayes Factors even suggested there was no association. The lack of association between behavioral and self-report measures is often observed in psychological research (Baumeister et al., 2007). However, this overall pattern of low convergence within and between measures together with the unexpected age-related findings pose the question of whether we measured the kind of goal focus that has been investigated in previous studies.

One might argue that the kind of goal focus as investigated in previous studies is not reflected in the simple tasks we used here. In this study, we developed tasks based on simple goals to make the study feasible for a large age range, starting in toddlerhood. Although this way all of the participants were able to master the tasks, it is not clear whether goal focus is the same for simple and complex goals and whether these tasks similarly reflect goal focus as previous measures. One observation that speaks against this lack of validity were the participants’ answers to some of the open-ended control questions. At least for the imitation choice task, there is anecdotal evidence that some of the participants based their decisions on whether “the path” or “the goal” was more important in the respective scenario (97 of the 225 participants who were asked what they had based their decisions on referred to “the path” and/or “the goal/result/endpoint”). These are post-hoc explanations that might not reflect the participants’ thoughts during the task. The fact that the results did not reveal age-related differences in this task (although some tendencies are visible) might either indicate that the concept of goal focus has a different function in simple goals or be a result of the decision on how to code the data. The coding followed the procedure by Elsner and Pfeifer (2012), considering the first means and the first outcome on which participants acted. This method excludes further object explorations and play shown by some of the participants. However, this way of coding also ignored decisions in which participants changed their minds, which might be more informative regarding the participants’ goal focus (e.g., putting the manikin into the boat, then realizing the boat went to the bed instead of to the chair, and then putting the manikin into the car and go to the chair). Due to difficulties to differentiate these kinds of behaviors as objectively as possible across the different age groups in our coding scheme, we kept the original coding. Because of the methodological drawbacks of using simple goals geared towards very young children and the unexpected findings, we conducted a follow-up study with a larger, adult sample, to inspect the verbal measures of the study in more detail.

## Study 2

Study 2 combined the measures of Study 1 with another established measure of goal focus that is based on more complex goals (Freund et al., 2010). With this, we aimed to investigate whether we can replicate previous findings on the association of age and goal focus and how this measure relates to the verbal measures used in Study 1 in an online setting. The study conformed to the ethical standards of the 1964 Helsinki declaration and its later amendments and the regulations of the ethics committee of the Faculty of Arts and Social Sciences at the University of Zurich. The study was preregistered at https://osf.io/pg4kv. At this time, approximately two thirds of the data had already been collected. However, none of the authors had access to the data before the preregistration. The materials and the anonymized data are available at https://osf.io/zsr28/.

### Methods

#### Sample

Participants were recruited via Respondi (https://www.respondi.com/, a German online panel) and reimbursed according to Respondi’s regulations. To control the reliability of the data, we included some quality check questions (e.g., asking for both age and date of birth in different places of the questionnaire) and participants who failed these checks were excluded from the analysis. The final sample consisted of a total of 1550 participants, of which 515 participants were between 17 and 35 years, 505 participants were between 36 and 64 years, and 530 participants were 65 years and older (see Table 5 for more details on the sample).

**Table 5:** Sample characteristics of study 2

Subsample	*n*	*M* age in years	*SD* age in years	% female	Distribution by country
17–35 years	515	27.77	4.80	50.3	33% Swiss, 31.7% German, 35.3% Austrian
36–64 years	505	50.39	8.03	49.3	33.3% Swiss, 33.7% German, 33% Austrian
65 years and older	530	70.64	4.33	45.8	36.8% Swiss, 31.9% German, 31.3% Austrian

#### Procedure and measures

This online study was part of a larger study. After having given informed consent, participants provided demographic data (age, gender, nationality, marital status, education, income, life satisfaction, and subjective health) and then completed questionnaires unrelated to this study. Next, they completed the measures for this study (either first the Study 1 measures and then the Freund et al. measure (Freund et al., 2010), or the other way around). It is important to note that we did not change the order of the Study 1 measures. The Study 1 measures included the action descriptions, the thinking exercise, and the motto items. They were identical to the ones described in Study 1. The Freund et al. measure described four goals with 10 statements each, five formulated in terms of the means, and five in terms of the ends of the respective goal (see Supplemental Material, Text S3). Participants had to choose five of the ten statements that best described the goal in their opinion. The variable of interest was the mean value of means statements chosen across the four goals.

#### Data preparation and analysis

We prepared the data of the action descriptions, thinking exercise, and motto items according to the procedures mentioned in Study 1. For the Freund et al. measure, the number of “how” statements chosen for each goal was counted and averaged across the four goals (score from 0 to 5), as described in the original paper (Freund et al., 2010). For data analysis, we used the ordinal package (Christensen, 2018) in R (R Core Team, 2018) to compute ordinal and logistic regressions with the clm function to predict the different DVs (scores of the respective measure) from age. Because the data of the thinking exercise seemed to include nonlinear age effects, we also explored the quadratic effect of age in a logistic regression model. To test for convergence of the different measures, we ran Kendall’s correlations with Holm’s correction for the ordinal measures (action descriptions, motto items, and Freund et al. measure) and a logistic regression to predict the thinking exercise from the other measures. For the logistic regressions as well as the correlations, we additionally report Bayes Factors. Also, we report the inter-item correlations for the action descriptions and the items of the Freund et al. measure in the Supplemental Material (Table S7).

### Results

#### Age-related differences in goal focus

To correct for multiple testing, we only report findings as significant with *p* < 0.01, because of the five dependent variables we tested (Bonferroni’s correction). In the action descriptions, age significantly negatively predicted the number of process statements chosen (b* = − 0.48, *SE* = 0.05, *p* < 0.001). Post-hoc pairwise comparisons of the three age groups with Tukey adjustment showed significant differences between young and middle-aged adults (*p* < 0.001), young and older adults (*p* < 0.001), and middle-aged and older adults (*p* < 0.001). This is in line with the analysis of the total sample in Study 1, where older adults chose fewer process statements compared to adolescents and young adults.

In the thinking exercise, overall, participants chose the “why” thinking exercise more often than the “how” thinking exercise (63.5% vs. 36.5%). The logistic regression to predict participants’ decisions in the thinking exercise from age showed a significant linear effect (b* = − 0.18, *SE* = 0.05, *p* < 0.001), indicating that with age participants were more likely to choose thinking about the outcomes. This was supported by the Bayes Factor indicating that an age effect unequal to zero was approximately nine times more likely than an effect equal to zero (BF = 9.49). Once the quadratic effect of age was included in the logistic regression, both the linear (b* = − 0.20, *SE* = 0.05, *p* < 0.001) and the quadratic effect (b* = − 0.23, *SE* = 0.07, *p* = 0.002) were significant. A chi-square test to compare both models revealed that that model including the quadratic effect of age fit the data better; *χ*^2^(1) = 10.02, *p* = 0.002. Post-hoc pairwise group comparisons with Tukey adjustment revealed significant differences between young and older adults (*p* = 0.001), and middle-aged and older adults (*p* < 0.001), but not young and middle-aged adults (*p* = 0.60).

As for the motto items, all three age groups were more likely to agree than disagree with the process focus motto (“The path is the goal”), as indicated by mean values and medians above the scale midpoint. Age significantly predicted the agreement to the first motto (b* = 0.19, *SE* = 0.05, *p* < 0.001), but did not reach significance for the outcome motto (“It does not matter how I do it, the main thing is to get to the goal”; b* = − 0.11, *SE* = 0.05, *p* = 0.01). Post-hoc pairwise comparisons of the three age groups with Tukey adjustment for the first motto showed significant differences between young and middle-aged adults (*p* = 0.001), and young and older adults (*p* < 0.001), but not middle-aged and older adults (*p* = 0.96).

Regarding the Freund et al. (2010) measure, age was significantly positively associated with the number of process statements chosen (b* = 0.21, *SE* = 0.04, *p* < 0.001). Post-hoc pairwise comparisons of the three age groups with Tukey adjustment revealed a significant difference between young and old adults (*p* < 0.001), but not young and middle-aged (*p* = 0.11), and middle-aged and older adults (*p* = 0.05). Plots to visualize the data are provided in the Supplemental Material, Figure S2.

#### Convergence of measures

Kendall’s correlations for the ordinal measures are depicted in Table 6. Significant correlations were obtained for the action descriptions and the Freund et al. (2010) measure (*τ* = 0.10, *p* < 0.001, BF_10_ = 4.817e^6^), as well as between the two motto items (*τ* = -0.21, *p* < 0.001, BF_10_ = 8.506e^31^). In the logistic regression to predict the thinking exercise from the other measures, the action descriptions (b* = 0.18, *SE* = 0.05, *p* < 0.001, BF_10_ = 7.23), and the second motto item (b* = 0.16, *SE* = 0.06, *p* = 0.004, BF_10_ = 1.52) emerged as significant predictors based on the frequentist analysis. However, for the second motto item, the Bayes factor did not indicate support for convergence (see Table 7).

**Table 6:** Convergence of measures: Results from Kendall’s correlations

DVs	Action descriptions	Motto 1	Motto 2
Motto 1	− 0.04, *p* = 0.61, BF_10_ = 0.33	–	
Motto 2	< − 0.001, *p* > 0.99, BF_10_ = 0.03	− **0.21,** ***p* < 0.001, BF**_**10**_** = 8.506e**^**31**^	–
Freund et al. (2010) measure	**0.10,** ***p* < 0.001, BF**_**10**_** = 4.917e**^**6**^	− 0.01, *p* > 0.99, BF_10_ = 0.04	− 0.02, *p* > 0.99, BF_10_ = 0.06

*p* values are Holm-adjusted, and significant values are printed in bold. Motto 1 = “The path is the goal,” Motto 2 = “It does not matter how I do it, the main thing is to get to the goal.”

**Table 7:** Convergence of measures: Results from the logistic regression to predict the thinking exercise from the other tasks

Freund et al. measure	Action descriptions	Motto 1	Motto 2
0.09 (0.05) (*p* = 0.09, BF_10_ = 0.10)	**0.18 (0.05) (*p* < 0.001, BF**_**10**_** = 7.23)**	0.03 (0.06) (*p* = 0.54, BF_10_ = 0.03)	**0.16 (0.06) (*p* = 0.004, BF**_**10**_** = 1.52)**

Standardized regression coefficients with SEs in parentheses. Significant results (after correcting for multiple testing) are printed in bold

### Discussion

The results of this online study are partly in line with Study 1 and Freund et al. (2010). On the one hand, the findings replicated those of Study 1 that older adults chose fewer process statements than younger adults in the action descriptions. On the other hand, the findings also replicated those of Freund et al. (2010) that older adults chose more process statements than younger adults in their ten-statements measure. Different from Study 1, older adults were more likely to choose to think about the outcomes of goal pursuit and also agreed more with the motto “The path is the goal” relative to younger adults. These differences in findings might be due to the larger sample size in Study 2 as well as the approach to test for linear effects of age across adulthood.

The findings of Study 2 seem to partly contradict one another, with some pointing towards a stronger process focus, and others towards a stronger outcome focus with age. This is especially surprising in the case of the action descriptions and Freund et al.’s ten-statement measure, as they were very similar in structure (i.e., choosing one of two vs. choosing five of ten statements). However, note that these two measures differ in that the action descriptions refer to simple actions (e.g., brushing teeth), whereas the Freund et al. measure refers to more complex, higher-order goals (e.g., quitting smoking). This difference in the level of concrete actions to comparatively higher-order goals might have led to diverging age-related effects.

Regarding the convergence of the different measures, we found an association between the thinking exercise and the action descriptions, again replicating Study 1. Further, the action descriptions correlated positively with the Freund et al. measure, which was surprising given their diverging age correlations. This suggests that these measures might share a certain portion of variance that is unrelated to age. Regarding the inter-item correlations, the action descriptions showed on average a lower correlation than in study one (0.240 vs. 0.315) but still showed some degree of consistency, whereas the inter-item correlations of the Freund et al. measure were relatively low (see Supplemental Material, Table S7), questioning the reliability and validity of this measure.

## General discussion

We presented two studies that represent a first attempt to empirically investigate goal focus across the entire lifespan from young childhood into old age. The studies highlight some of the challenges of adopting an entire lifespan approach instead of focusing on one age phase only.

Using a multi-measures approach, we operationalized goal focus using several behavioral tasks and self-report measures. We did so to capture the construct in all age groups and different expressions of goal representations amenable to young children as well as adults. However, this approach proved problematic as the measures did not converge. This is a general problem known also in other areas of psychology such as the lack of convergence of different measures of inhibition and interference in a lifespan approach (Friedman & Miyake, 2004).

Turning to the specific challenge of using a lifespan approach, we found very little convergence of measures across all age groups in Study 1, but some indication that convergence might be age-dependent (see Supplemental Material, Tables S4–S6). Together with the complex associations among the measures in Study 2 and their association with age, as well as the low consistency of some measures, this raises the question of whether goal focus constitutes a uniform construct across the lifespan. As goal focus is defined on the phenomenological level (i.e., whether the means or ends are more salient to a person in a given goal), the underlying processes and functions of why the phenomenon occurs might differ substantially across the lifespan, and potentially also between very simple and more complex goals (see Moersdorf et al., 2022b). This potential variety in underlying processes poses the conceptual question of whether the phenomenon subsumed as goal focus should be considered one construct and if so, whether it might have multiple facets. For instance, there might be a facet of goal focus relating to simple, short-term goals. Within this facet, goal focus might be mainly driven by a person’s skills and experiences with goal-relevant actions. In complex, longer-term goals this might look quite different. There, goal focus might be driven by differences in goal orientation, as some work in adults has shown (Mustafić and Freund, 2012).

Even if the same construct of goal focus or facet of it could theoretically be observed in different age groups, it remains an open question whether the different measures we used captured it. The results of the present studies seem to suggest that this is not the case for our operationalizations of goal focus. For instance, the measures we used in the current studies varied in how simple or complex the goals were (e.g., from brushing teeth in the action descriptions to quitting smoking in the goal focus questionnaire by Freund et al., 2010). The complexity might have impacted whether the participants focused more on the means or outcomes. In simple action descriptions, older adults might have focused more on the gist, that is, the outcome in this task (Brainerd and Reyna, 2004). The small portion of shared variance between these two measures might represent goal focus independent of goal complexity. In contrast, the portion of the variance in both measures associated with age might reflect the aspects of goal focus that depend on the complexity of a given goal. Thus, one venue for further exploration is to systematically investigate differences in the complexity of goals.

However, low goal complexity cannot explain the negative association of the thinking exercise and age, because in the thinking exercise, the goal was to have a good vacation which is much more complex than the goals in the action descriptions. Consequently, there might be other measurement-related factors at play that influence the participants’ goal focus ratings, such as the method of the assessment itself, or how well the participants can relate to the respective goal. In addition, developmentally relevant factors such as changes in temporal proximity to certain goals and the cognitive capacities to process goals across the lifespan might impact goal focus ratings. Further research is needed to address the questions of which assessment methods work best to assess goal focus reliably and validly, whether there are stable, age-related differences in goal focus that can be detected despite goal-related and interindividual variation, and whether different facets of goal focus need to be differentiated (e.g., based on goal complexity).

An alternative approach to the lifespan study of goal focus would have been to create a task that converges with one of the established measures for one age group first, and then apply it stepwise to other phases of the lifespan. However, convergence within one age group does not necessarily imply convergence for other age groups. Similarly, this approach does not guarantee measurement invariance of the new measure across age groups. Furthermore, low convergence of measures does not necessarily mean that they index different constructs; instead, they might measure different facets of the same construct. In our approach, we aimed to cover goal focus broadly and, therefore, constructed multiple measures based on face validity.

Because we find neither clear evidence for nor against the hypothesized age-related differences in goal focus, we deem it crucial to deepen our understanding of the construct in a way that enables us to define the boundaries of the construct and its association with age. We are convinced that this knowledge will also be essential to improve our comprehension of the adaptiveness of goal focus during goal pursuit.

## Conclusion

This paper aimed to present a first step to study goal focus across the entire lifespan by using a multimethodological approach, which encompassed eye tracking, behavioral, and verbal measures. The findings we report are mixed and reflect the challenges an entire lifespan approach entails on different levels. Especially the issues of convergence of measures and measurement construction across age groups pose a veritable challenge to the valid and reliable investigation of constructs across the entire lifespan. Despite these challenges, research comprising the entire lifespan has a unique value for understanding developmental processes that render it worthwhile to do so.

## Supplementary Information

Below is the link to the electronic supplementary material.

Supplementary file1 (DOCX 581 KB)

## Data Availability

All data and files related to Study 2 are available at OSF (https://osf.io/zsr28/). Data from Study 1 cannot be shared publicly because the consent form did not include a data-sharing statement allowing online sharing. These data are available only upon reasonable request.

## Footnotes

1 Overimitation refers to the phenomenon that people do not only imitate action steps that are causally relevant to achieve a certain outcome but also causally irrelevant steps.

2 Normative criticism describes a type of protesting behavior that is based on a normative argumentation (e.g., “This is not how this is/should be done! You have to do it like this.”).

3 The sample size was determined based on feasibility considerations as well as group size conventions. We refrained from doing a power analysis because there was no previous literature to estimate effect sizes from. However, we report post-hoc sensitivity analyses for the main results in the Supplemental Material (Table S2).
