# Supplementary material for: What do you focus on? An investigation of goal focus from childhood to old age
Source: Psychol Res. 2023 Feb 21;87(7):2120–37. doi: 10.1007/s00426-023-01804-0 (PMC10457216; doi:10.1007/s00426-023-01804-0)
Supplement: Supplementary file 1 — Supplementary file1 (DOCX 581 KB) [file 426_2023_1804_MOESM1_ESM.docx]

# Supplemental Material

Overview 2

Study 1 2

Table S1. List of Presented Goal Focus Trials and Control Trials in Gaze Allocation Task 2

Table S2. Post-Hoc Sensitivity Analyses for Overall Group Differences 3

Table S3. Inter-Item Correlations for the Gaze Allocation Task, the Imitation Choice Task, and the Action Descriptions 3

Figure S1. Plots of All Goal Focus Measures by Age Group 5

Text S1. Convergence of Measures Separately for the Age Groups 6

Table S4. Convergence of Measures by Age Group: Kendall’s Correlations Among the Behavioral Measures 6

Table S5. Convergence of Measures by Age Group: Kendall’s Correlations Among the Behavioral and Verbal Measures 7

Table S6. Convergence of Measures by Age Group: Results of Logistic Regression to Predict Thinking Exercise from Other Tasks 10

Text S2. Linear Effects of Age Across Adulthood 11

Study 2 12

Text S3. Instructions and Items of Freund et al. (2010) Measure 12

Table S7. Inter-Item Correlations for the Action Descriptions and the Items of the Freund et al. Measure 14

Figure S2. Plots of All Goal Focus Measures by Age (Group) 16

## Overview

In the following, we present the Supplemental Material as it occurs in the main manuscript (divided into Studies 1 and 2). First, we list the trials of the gaze allocation task and present the post-hoc sensitivity analyses. Then, we present plots of all goal-focus measures by age group. Next, we report the convergence of measures separate for age groups as obtained from analyzing the original data. Afterward, we show exploratory linear regression analyses of the original data where we only considered data from adults to compare it more easily to Study 2. Finally, we present the Freund et al. (2010) measure of Study 2 as well as the plots of all goal focus measures by age group.

## Study 1

### Table S1. List of Presented Goal Focus Trials and Control Trials in Gaze Allocation Task

| Goal focus trials | |  | Control trials | |
| --- | --- | --- | --- | --- |
| Means | Outcome |  | Moving | Static |
| Fold paper plane | Finished paper plane |  | Rolling ball | Static ball |
| Form playdough figure | Finished playdough figure |  | Falling bottle | Standing bottle |
| Put together styrofoam figure | Finished styrofoam figure |  | Falling leaf | Lying leaf |

### Table S2. Post-Hoc Sensitivity Analyses for Overall Group Differences

| Task | Type of analysis | *N* | Number of groups | Effect size | Non-centrality | Critical value |
| --- | --- | --- | --- | --- | --- | --- |
| Gaze allocation | ANOVA | 252 | 8 | *f* = 0.24 | *λ* = 14.77 | *F* = 2.05 |
| Behavioral preference | Kruskal Wallis tests | 293 | 8 | *f* = 0.22 | *λ* = 14.71 | *F* = 2.04 |
| Imitation choice |  | 276 | 8 | *f* = 0.23 | *λ* = 14.73 | *F* = 2.04 |
| Action descriptions |  | 211 | 6 | *f* = 0.25 | *λ* = 13.18 | *F* = 2.56 |
| Thinking exercise | Chi-square | 211 | 6 | *w* = 0.25 | *λ* = 12.83 | *χ*^2^ = 11.07 |
| Motto items | Kruskal Wallis tests | 211 | 6 | *f* = 0.25 | *λ* = 13.18 | *F* = 2.56 |

*Note.* All sensitivity analyses were calculated with an assumed power of .80 in G*Power. For the Kruskal-Wallis Tests, the sensitivity analysis for the ANOVA omnibus test was used.

### Table S3. Inter-Item Correlations for the Gaze Allocation Task, the Imitation Choice Task, and the Action Descriptions

| Task | Type of correlation | Correlation coefficient |
| --- | --- | --- |
| Gaze allocation task | across all items | .084 |
|  | paper – styro  paper – playdough  styro – playdough | .051  .094  .144 |
| Imitation choice task | across all items* | .221 |
|  | between blocks | .137 |
|  | pond – ramp  pond – board  ramp – board | .147  .130  .125 |
| Action descriptions | across all items | .315 |
|  | wash – lock  wash – brush  wash – ring  wash – eat  lock – brush  lock – ring  lock – eat  brush – ring  brush – eat  ring – eat | .186  .362  .352  .308  .339  .269  .219  .453  .383  .277 |

*Note.* Correlations were calculated according to Pearson for the gaze allocation task, according to Spearman for the imitation choice task, and based on Phi coefficients for the action descriptions; paper = depiction of a paper plane, styro = depiction of styrofoam figure, playdough = depiction of playdough figure; wash, lock, brush, ring, eat = items referring to the actions of washing clothes, locking the door, brushing the teeth, ringing a doorbell, and eating. *The correlations between all items are higher than between blocks because the former also include the correlations of the items within one block (pond 1 – pond 2: r = .596; board 1 – board 2: r = .598; ramp 1 – ramp 2: r = .707).

### Figure S1. Plots of All Goal Focus Measures by Age Group

### Text S1. Convergence of Measures Separately for the Age Groups

When looking at the convergence of measures separately for the different age groups, neither the correlations nor the logistic regressions indicate significant convergence among measures after correcting for multiple testing (none of the presented Holm-adjusted *p* values is < .05; Tables S3-S5). The Bayes Factors in Table S4 (relevant values printed in bold) indicate that the 13-17-year-olds and the 75-85-year-olds probably drove the tendency of a negative association between the action descriptions and the preference task in the overall convergence analysis. The same holds for the 18-25-year-olds and 35-45-year-olds regarding the two motto items. Also, Bayes Factors were indicating moderate to strong evidence for a positive association between the imitation choice task and the first motto item in the 13-17-year-olds, a negative association between the imitation choice task and the behavioral preference task in the 55-65-year-olds, and a negative association between the gaze allocation task and the action descriptions in the 75-85-year-olds. However, it should be noted that according to the frequentist analyses, none of these associations reaches significance. As to the association of the action descriptions and the thinking exercise, the Bayes Factors in Table S5 do not indicate any group that drove this effect.

### Table S4. Convergence of Measures by Age Group: Kendall’s Correlations Among the Behavioral Measures

| **Age group** |  | **Gaze allocation** | **Imitation choice** |
| --- | --- | --- | --- |
| **3.5-4.5** | Imitation choice | .03 (*n* = 24)  (*p* = .99, BF_10_ = 0.27 | - |
|  | Behavioral preference | .30 (*n* = 26)  (*p* = .40, BF_10_ = 2.29) | -.11 (*n* = 40)  (*p* = .99, BF_10_ = 0.33) |
| **6-7** | Imitation choice | .06 (*n* = 34)  (*p* >.99, BF_10_ = 0.25) | - |
|  | Behavioral preference | -.08 (*n* = 35)  (*p* >.99, BF_10_ = 0.27) | -.12 (*n* = 40)  (*p* >.99, BF_10_ = 0.36) |

*Note.* *p* values are Holm-adjusted within age group, only the youngest two age groups are shown.

### Table S5. Convergence of Measures by Age Group: Kendall’s Correlations Among the Behavioral and Verbal Measures

| **Age group** |  | **Gaze allocation** | **Imitation choice** | **Behavioral preference** | **Action descriptions** | **Motto 1** |
| --- | --- | --- | --- | --- | --- | --- |
| **10-11** | Imitation choice | -.01 (*n* = 27)  (*p* >.99, BF_10_ = 0.25) | - | - | - | - |
|  | Behavioral preference | -.03 (*n* = 29)  (*p* >.99, BF_10_ = 0.25) | .10 (*n* = 30)  (*p* >.99, BF_10_ = 0.31) | - | - | - |
|  | Action descriptions | .01 (*n* = 29)  (*p* >.99, BF_10_ = 0.24) | .21 (*n* = 30)  (*p* >.99, BF_10_ = 0.85) | -.10 (*n* = 32)  (*p* >.99, BF_10_ = 0.31) | - | - |
|  | Motto 1 | -.08 (*n* = 29)  (*p* >.99, BF_10_ = 0.29) | -.04 (*n* = 30)  (*p* >.99, BF_10_ = 0.25) | -.04 (*n* = 32)  (*p* >.99, BF_10_ = 0.24) | -.15 (*n* = 32)  (*p* >.99, BF_10_ = 0.46) | - |
|  | Motto 2 | .26 (*n* = 29)  (*p* >.99, BF_10_ = 1.57) | -.25 (*n* = 30)  (*p* >.99, BF_10_ = 1.44) | .04 (*n* = 32)  (*p* >.99, BF_10_ = 0.24) | -.05 (*n* = 32)  (*p* >.99, BF_10_ = 0.25) | .20 (*n* = 32)  (*p* >.99, BF_10_ = 0.80) |
| **13-17** | Imitation choice | .22 (*n* = 26)  (*p* >.99, BF_10_ = 0.83) | - | - | - | - |
|  | Behavioral preference | .04 (*n* = 27)  (*p* >.99, BF_10_ = 0.26) | .17 (*n* = 28)  (*p* >.99, BF_10_ = 0.53) | - | - | - |
|  | Action descriptions | .27 (*n* = 27)  (*p* >.99, BF_10_ = 1.60) | .04 (*n* = 28)  (*p* >.99, BF_10_ = 0.25) | **-.37 (*n* = 29)**  **(*p* = .64, BF_10_ = 10.82)** | - | - |
|  | Motto 1 | .08 (*n* = 27)  (*p* >.99, BF_10_ = 0.29) | .**40 (*n* = 28)**  **(*p* = .54, BF_10_ = 17.49)** | .27 (*n* = 29)  (*p* >.99, BF_10_ = 1.82) | -.07 (*n* = 29)  (*p* >.99, BF_10_ = 0.27) | - |
|  | Motto 2 | .20 (*n* = 27)  (*p* >.99, BF_10_ = 0.69) | -.12 (*n* = 28)  (*p* >.99, BF_10_ = 0.36) | -.18 (*n* = 29)  (*p* >.99, BF_10_ = 0.59) | < .01 (*n* = 29)  (*p* >.99, BF_10_ = 0.24) | -.22 (*n* = 29)  (*p* >.99, BF_10_ = 0.92) |
| **18-25** | Imitation choice | .11 (*n* = 45)  (*p* >.99, BF_10_ = 0.34) | - | - | - | - |
|  | Behavioral preference | -.21 (*n* = 46)  (*p* >.99, BF_10_ = 1.51) | -.10 (*n* = 48)  (*p* >.99, BF_10_ = 0.31) | - | - | - |
|  | Action descriptions | -.07 (*n* = 46)  (*p* >.99, BF_10_ = 0.24) | -.04 (*n* = 48)  (*p* >.99, BF_10_ = 0.20) | -.10 (*n* = 50)  (*p* >.99, BF_10_ = 0.31) | - | - |
|  | Motto 1 | .03 (*n* = 46)  (*p* >.99, BF_10_ = 0.20) | -.02 (*n* = 48)  (*p* >.99, BF_10_ = 0.19) | .11 (*n* = 50)  (*p* >.99, BF_10_ = 0.34) | -.05 (*n* = 51)  (*p* >.99, BF_10_ = 0.21) | - |
|  | Motto 2 | -.02 (*n* = 46)  (*p* >.99, BF_10_ = 0.19) | -.04 (*n* = 48)  (*p* >.99, BF_10_ = 0.20) | -.09 (*n* = 50)  (*p* >.99, BF_10_ = 0.28) | -.02 (*n* = 51)  (*p* >.99, BF_10_ = 0.19) | **-.35 (*n* = 51)**  **(*p* = .18, BF_10_ = 111.66)** |
| **35-45** | Imitation choice | -.01 (*n* = 27)  (*p* >.99, BF_10_ = 0.25) | - | - | - | - |
|  | Behavioral preference | .15 (*n* = 28)  (*p* >.99, BF_10_ = 0.44) | .33 (*n* = 29)  (*p* >.99, BF_10_ = 4.97) | - | - | - |
|  | Action descriptions | .07 (*n* = 28)  (*p* >.99, BF_10_ = 0.28) | .17 (*n* = 29)  (*p* >.99, BF_10_ = 0.54) | .25 (*n* = 30)  (*p* >.99, BF_10_ = 1.44) | - | - |
|  | Motto 1 | .07 (*n* = 28)  (*p* >.99, BF_10_ = 0.28) | .01 (*n* = 29)  (*p* >.99, BF_10_ = 0.24) | .19 (*n* = 30)  (*p* >.99, BF_10_ = 0.67) | .15 (*n* = 30)  (*p* >.99, BF_10_ = 0.45) | - |
|  | Motto 2 | -.23 (*n* = 28)  (*p* >.99, BF_10_ = 1.00) | .13 (*n* = 29)  (*p* >.99, BF_10_ = 0.38) | -.09 (*n* = 30)  (*p* >.99, BF_10_ = 0.30) | -.09 (*n* = 30)  (*p* >.99, BF_10_ = 0.30) | **-.50 (*n* = 30)**  **(*p* = .08, BF_10_ = 322.77)** |
| **55-65** | Imitation choice | .09 (*n* = 27)  (*p* >.99, BF_10_ = 0.30) | - | - | - | - |
|  | Behavioral preference | .19 (*n* = 28)  (*p* >.99, BF_10_ = 0.64) | **-.36 (*n* = 30)**  **(*p* = .74, BF_10_ = 10.03)** | - | - | - |
|  | Action descriptions | -.18 (*n* = 29)  (*p* >.99, BF_10_ = 0.59) | .16 (*n* = 30)  (*p* >.99, BF_10_ = 0.49) | -.11 (*n* = 31)  (*p* >.99, BF_10_ = 0.33) | - | - |
|  | Motto 1 | -.15 (*n* = 29)  (*p* >.99, BF_10_ = 0.45) | -.03 (*n* = 30)  (*p* >.99, BF_10_ = 0.24) | .09 (*n* = 31)  (*p* >.99, BF_10_ = 0.30) | -.12 (*n* = 32)  (*p* >.99, BF_10_ = 0.36) | - |
|  | Motto 2 | .14 (*n* = 29)  (*p* >.99, BF_10_ = 0.41) | -.03 (*n* = 30)  (*p* >.99, BF_10_ = 0.24) | .19 (*n* = 31)  (*p* >.99, BF_10_ = 0.69) | -.02 (*n* = 32)  (*p* >.99, BF_10_ = 0.23) | -.08 (*n* = 32)  (*p* >.99, BF_10_ = 0.28) |
| **75-85** | Imitation choice | -.09 (*n* = 24)  (*p* >.99, BF_10_ = 0.31) | - | - | - | - |
|  | Behavioral preference | .13 (*n* = 30)  (*p* >.99, BF_10_ = 0.38) | -.18 (*n* = 31)  (*p* >.99, BF_10_ = 0.61) | - | - | - |
|  | Action descriptions | **-.38 (*n* = 30)**  **(*p* = .54, BF_10_ = 15.38)** | -.10 (*n* = 31)  (*p* >.99, BF_10_ = 0.31) | **-.33 (*n* = 37)**  **(*p* = .64, BF_10_ = 11.65)** | - | - |
|  | Motto 1 | .12 (*n* = 30)  (*p* >.99, BF_10_ = 0.36) | .15 (*n* = 31)  (*p* >.99, BF_10_ = 0.46) | .13 (*n* = 37)  (*p* >.99, BF_10_ = 0.40) | -.13 (*n* = 37)  (*p* >.99, BF_10_ = 0.40) | - |
|  | Motto 2 | -.07 (*n* = 30)  (*p* >.99, BF_10_ = 0.27) | -.06 (*n* = 31)  (*p* >.99, BF_10_ = 0.26) | .15 (*n* = 37)  (*p* >.99, BF_10_ = 0.49) | .17 (*n* = 37)  (*p* >.99, BF_10_ = 0.62) | -.17 (*n* = 37)  (*p* >.99, BF_10_ = 0.62) |

*Note.* *p* values are Holm-adjusted within age group, only the age groups with verbal measures (from 10 years onward) are shown.

### Table S6. Convergence of Measures by Age Group: Results of Logistic Regression to Predict Thinking Exercise from Other Tasks

| **Age group** | **Gaze allocation** | **Behavioral preference** | **Imitation choice** | **Action descriptions** | **Motto 1** | **Motto 2** |
| --- | --- | --- | --- | --- | --- | --- |
| **10-11**  (*n* = 27) | -0.14(0.52)  (*p* = .78, BF_10_ = 0.20) | -0.04(0.53)  (*p* = .95, BF_10_ = 0.19) | -0.85(0.60)  (*p* = .15, BF_10_ = 0.53) | 1.34(0.77)  (*p* = .08, BF_10_ = 0.87) | 0.14(0.47)  (*p* = .77, BF_10_ = 0.20) | -0.11(0.47)  (*p* = .82, BF_10_ = 0.20) |
| **13-17**  (*n* = 26) | 0.20(0.51)  (*p* = .69, BF_10_ = 0.21) | -0.33(0.60)  (*p* = .58, BF_10_ = 0.23) | -0.16(0.48)  (*p* = .74, BF_10_ = 0.21) | -0.29(0.57)  (*p* = .61, BF_10_ = 0.22) | 0.34(0.58)  (*p* = .56, BF_10_ = 0.23) | 0.69(0.50)  (*p* = .17, BF_10_ = 0.51) |
| **18-25**  (*n* = 45) | -0.17(0.32)  (*p* = .59, BF_10_ = 0.17) | -0.66(0.37)  (*p* = .07, BF_10_ = 0.74) | -0.32(0.30)  (*p* = .29, BF_10_ = 0.26) | 0.61(0.36)  (*p* = .08, BF_10_ = 0.66) | -0.05(0.48)  (*p* = .91, BF_10_ = 0.15) | -0.43(0.49)  (*p* = .38, BF_10_ = 0.22) |
| **35-45**  (*n* = 27) | 1.57(1.22)  (*p* = .20, BF_10_ = 0.44) | -1.57(1.69)  (*p* = .35, BF_10_ = 0.30) | 0.73(0.89)  (*p* = .41, BF_10_ = 0.27) | 0.71(1.24)  (*p* = .57, BF_10_ = 0.23) | 3.10(1.75)  (*p* = .08, BF_10_ = 0.93) | 1.09(0.78)  (*p* = .17, BF_10_ = 0.50) |
| **55-65**  (*n* = 27) | -0.03(0.65)  (*p* = .97, BF_10_ = 0.19) | 0.58(0.63)  (*p* = .36, BF_10_ = 0.29) | -1.15(1.28)  (*p* = .37, BF_10_ = 0.29) | 2.05(1.11)  (*p* = .07, BF_10_ = 1.04) | 0.90(0.92)  (*p* = .33, BF_10_ = 0.31) | -0.22(0.55)  (*p* = .68, BF_10_ = 0.21) |
| **75-85**  (n = 24) | 0.09(0.80)  (*p* = .91, BF_10_ = 0.21) | 0.66(0.64)  (*p* = .30, BF_10_ = 0.35) | 0.90(0.67)  (*p* = .18, BF_10_ = 0.50) | 1.20(0.79)  (*p* = .13, BF_10_ = 0.66) | -0.04(0.41)  (*p* = .92, BF_10_ = 0.21) | 0.30(0.51)  (*p* = .56, BF_10_ = 0.24) |

*Note.* Standardized regression coefficients with SEs in parentheses, only the age groups with verbal measures (from 10 years onward) are shown.

### Text S2. Linear Effects of Age Across Adulthood

Because of these mainly unexpected findings, we decided to explore the adult subsample of our data to see whether there were any linear trends in our measures that would support previous findings of an increasing process focus with age. Therefore, we ran a linear (gaze allocation task), a logistic (thinking exercise), and ordinal regressions (preference task, imitation choice task, action descriptions, and motto items) with age as a continuous variable. We found a significant negative effect of age on the relative looking time towards process images, indicating that older adults looked relatively shorter at the process images compared to younger adults (*b** = -0.21, *SE* = 0.09, *p* = .02). Regarding the ordinal regressions, age did not significantly predict whether the process or outcome objects were picked in the preference task (*b** = -0.10, *SE* = 0.17, *p* = .56) or whether the process or outcome was imitated in the imitation choice task (*b** = 0.28, *SE* = 0.17, *p* = .10).

However, age significantly predicted the number of process statements chosen in the action descriptions (*b** = -0.49, *SE* = 0.16, *p* = .003), and the agreement to the two motto items (*b** = 0.34, *SE* = 0.16, *p* = .04; *b** = -0.32, *SE* = 0.16, *p* = .047). In the thinking exercise, age did not predict whether the process or outcome exercise was chosen (*b** = -0.15, *SE* = 0.20, *p* = .44). To summarize, the older participants were, the fewer process statements they chose in the action descriptions, the more they agreed to the motto “The path is the goal”, and the less they agreed to the motto “It does not matter how I do it, the main thing is to get to the goal.”

## Study 2

### Text S3. Instructions and Items of Freund et al. (2010) Measure

“Activities can be described in different ways. You may describe “hiking” as “increasing my fitness” or as “enjoying nature.” Both of these descriptions are correct – they just refer to different aspects of the activity. Below, you will find four activities followed by 10 descriptions. **Please select** **5 statements** for each of the activities that, for you personally, describe the activity best. Note that some of the activities might not apply to things you are currently doing. For these activities, consider how you would describe them if you were actually doing them.”

**Activity: To build a house**

1. Settling down (o)
2. Owning a house (o)
3. Finding a good location (p)
4. Planning the layout of the rooms (p)
5. Building a “nest” (o)
6. Having more space (o)
7. Hiring an architect (p)
8. Getting financial advice (p)
9. Having full freedom to remodel the house in the future (o)
10. Having plans drawn up (p)

**Activity: Maintain a relationship**

1. spend time together (p)
2. talk to each other (p)
3. increase the quality of the relationship (o)
4. have somebody to trust (o)
5. give partner small presents (p)
6. avoid a separation (o)
7. be attentive to partner (p)
8. have somebody by my side (o)
9. reduce loneliness (o)
10. listen to the partner (p)

**Activity: Quit smoking**

1. identify substitutes (e.g., chewing gum) (p)
2. reduce toxins in my body (o)
3. spend time with non-smokers (p)
4. be able to breathe more easily (o)
5. save money (o)
6. throw away ashtrays and cigarette lighters (p)
7. do something for my health (o)
8. not being addicted (o)
9. not buying or having cigarettes around (p)
10. think about potential negative consequences of smoking (p)

**Activity: Organize a high school reunion**

1. finding out how my classmates “turned out” (o)
2. planning the schedule of the reunion (p)
3. designing the invitations (p)
4. finding a good venue (p)
5. meeting former classmates and teachers (o)
6. reliving the past for a brief period of time (o)
7. exchanging different perspectives on high school (o)
8. getting recognition from former classmates (o)
9. sending out invitations (p)
10. researching addresses of former classmates (p)

*Note.* o and p indicate outcome and process statements.

### Table S7. Inter-Item Correlations for the Action Descriptions and the Items of the Freund et al. Measure

| Task | Type of correlation | Correlation coefficient |
| --- | --- | --- |
| Action descriptions | across all items | .240 |
|  | wash – lock  wash – brush  wash – ring  wash – eat  lock – brush  lock – ring  lock – eat  brush – ring  brush – eat  ring – eat | .238  .291  .356  .181  .278  .203  .195  .261  .233  .162 |
| Freund et al. measure | across all items | .114 |
|  | house – relationship  house – smoking  house – reunion  relationship – smoking  relationship – reunion  smoking – reunion | .124  .109  .222  .021  .083  .126 |

*Note.* The correlation coefficients were based on Phi coefficients for the action descriptions and Spearman correlations for the Freund et al. measure.

### Figure S2. Plots of All Goal Focus Measures by Age (Group)
